# A comparative study of *Helicobacter pylori* infection in hamsters experimentally infected with liver flukes *Opisthorchis felineus*, *Opisthorchis viverrini*, or *Clonorchis sinensis*

**DOI:** 10.1038/s41598-021-87446-x

**Published:** 2021-04-08

**Authors:** Maria Y. Pakharukova, Oxana Zaparina, Sung-Jong Hong, Banchob Sripa, Viatcheslav A. Mordvinov

**Affiliations:** 1 Institute of Cytology and Genetics, Siberian Branch of Russian Academy of Sciences, 10 Lavrentiev Ave., Novosibirsk, Russia 630090; 2 Department of Natural Sciences, Novosibirsk State University, 2 Pirogova Str., Novosibirsk, Russia 630090; 3 Department of Medical Environmental Biology, Chung-Ang University College of Medicine, Seoul, Republic of Korea; 4 WHO Collaborating Centre for Research and Control of Opisthorchiasis (Southeast Asian Liver Fluke Disease), Tropical Disease Research Center, Department of Pathology, Faculty of Medicine, Khon Kaen University, Khon Kaen, 40002 Thailand

**Keywords:** Microbiology, Zoology, Diseases, Pathogenesis

## Abstract

*Helicobacter pylori* causes a wide range of human diseases including cancer. Carcinogenic foodborne trematodes *Opisthorchis viverrini*, *Clonorchis sinensis,* and *O*. *felineus* might promote transmission and spread of *H*. *pylori* infection in the definitive mammalian host, which in turn might contribute to the liver fluke-associated malignancy. Our objectives were to find out whether liver flukes *O*. *felineus*, *O*. *viverrini*, and *C*. *sinensis* are carriers of *Helicobacter pylori* and to determine whether *H*. *pylori* is present in feces, bile, and stomach samples from the experimentally infected hamsters. We found that liver flukes are not reservoirs of *H*. *pylori*. Nevertheless, the prevalence of *H*. *pylori* and the *H*. *pylori ureA* gene copy number were significantly elevated after the infection. Overall, although the liver flukes *O*. *felineus*, *C*. *sinensis*, and *O*. *viverrini* are not reservoirs of *H*. *pylori*, the infection with the liver flukes significantly modifies the biliary and gut microbiota by increasing *H*. *pylori* abundance. This may be a feature of any liver fluke pathogenesis that have not previously been taken into account. Our findings appear to be novel in terms of comparative assessment of the host microbiota and *Helicobacter* abundance during epidemiologically important liver fluke infections.

## Introduction

Three epidemiologically significant food-borne trematodes *Opisthorchis felineus*, *O*. *viverrini*, and *Clonorchis sinensis* contain in their life cycle two intermediate hosts (freshwater gastropods and cyprinids) and one final host (fish-eating mammals, including humans). Despite the similarity in the life cycle and pathogenicity for the final host, the liver flukes also have marked differences. The differences primarily concern their geographic ranges, which do not overlap, and their different climatic zones. In particular, *O*. *felineus* is endemic in Western Siberia and Eastern Europe; *O*. *viverrini* in Southeast Asia, predominantly in Thailand, and Laos; and *C*. *sinensis* is endemic in China and South Korea. It is important to note that the main reservoir of *O*. *felineus* in Western Siberia is wild fish-eating animals, such as feral carnivores^1,2^. It is believed that the zoonotic cycle of *O*. *viverrini* has largely disappeared, having been replaced by a predominantly anthropogenic cycle^1^. *Clonorchis* *sinensis* geographic areas hold an intermediate position, with many native and domestic reservoir animal hosts and with high levels of human fecal contamination of the environment playing a considerable role in the transmission cycle^3^.

There are also differences in the carcinogenic potential of trematodes. In particular, *O*. *viverrini* and *C*. *sinensis* are recognized as the first group of biological carcinogens (Group 1A), and the highest incidence of cholangiocarcinoma (CCA) associated with the liver fluke infection is observed in anthropogenic areas of infection in Thailand and Lao PDR^4^. *Opisthorchis* *felineus* carcinogenicity is not as pronounced as that of *O*. *viverrini* and *C*. *sinensis*^2,5^, and this species is recognized as a potentially dangerous agent (Group 2B)^4^.

The mechanisms of CCA initiation under the influence of the liver flukes are investigated insufficiently; in particular, it is not known whether the differences in the carcinogenicity among these three related liver fluke species are associated with differences in the trematode microbiota^6^. Taking into account the climatic differences, nonoverlapping geographic ranges, the presence of a free-living aquatic life stage, and different natural reservoirs of the liver flukes, there should be differences in the microbiome of these three epidemiologically significant species.

One of the hypotheses explaining the differences in carcinogenicity among helminths is that their microbiota influences the pathogenesis of infection^7–9^. In particular, the relation between *Helicobacter pylori* infection and liver pathologies has been discussed for many years. *Helicobacter* *pylori*, as a biological carcinogen, can be a risk factor for CCA in patients with opisthorchiasis. The high prevalence of *Helicobacter* spp. in hamsters and the associated prevalence of hepatobiliary disorders also raise the question whether *Helicobacter* spp. have been acting as tumor promoters in the liver of hamsters infected with the liver flukes.

There is growing evidence that *H*. *pylori* has an important role in extragastric cancers^8^. An association was found between an increased risk of bile duct cancer in humans and the presence of *Helicobacter* bacteria^10^. In that study, *H*. *pylori* was detected significantly more frequently in the gallbladder of the malignant group than in the benign group. In addition, *H*. *pylori* was detected in the liver tissues and gallstones of patients with CCA and cholelithiasis, respectively. It has been hypothesized that *O*. *viverrini* is a reservoir of *H*. *pylori* and that this liver fluke is a vector for *H*. *pylori* in humans^11,12^.

Currently, there are some data on microbiomes in the liver flukes, but these data differ significantly in microbial composition. In addition, these results have been obtained using conventional animals in experiments that varied in design. Thus, the questions whether any species of epidemiologically significant Opisthorchiidae trematodes can serve as reservoirs for *H*. *pylori* and whether mammals become infected with these bacteria during infection with the liver flukes remain open.

To answer these questions, the following investigation was planned: (i) infection with *O*. *felineus*, *O*. *viverrini*, or *C*. *sinensis* metacercariae at an animal facility free from specific pathogens; (ii) a comparative analysis of *H*. *pylori* content in various samples from the experimental animals.

## Materials and methods

### Animals

Seventy-two male golden Syrian hamsters (*M*. *auratus*) from the Specific Pathogen-Free (SPF) Animal Facility of the ICG SB RAS were used for this study. The SPF Facility tests the laboratory animals for *Helicobacter* spp. pathogens once a year in accordance with FELASA guidelines for the health monitoring of laboratory animals.

For collecting *O*. *felineus* metacercariae, a naturally infected freshwater fish (*Leuciscus idus*) was net-caught in the Ob River near Novosibirsk (Western Siberia, Russia) by research assistant Viktor Antonov (ICG SB RAS) without the use of chemicals. *Opisthorchis* *felineus* metacercariae were extracted as described previously^13,14^. *Clonorchis* *sinensis* and *O*. *viverrini* metacercariae were extracted from naturally infected freshwater fish (Seoul, Republic of Korea, and Khon Kaen, Thailand, respectively). After several washes with normal saline, metacercariae were identified under a light microscope.

All the procedures were in compliance with EU Directive 2010/63/EU for animal experiments. Study design protocols and standard operating procedures (concerning the hamsters and the fish) were approved by the Committee on the Ethics of Animal Experiments at the ICG SB RAS (permit number 42 of 25 May 2018).

### Experimental design

Seventy-two male golden Syrian hamsters were used in this study. We applied appropriate randomisation strategy (blocking) to control the possible variables, such as potential *H*. *pylori* infection among experimental animals. We took into account nuisance variables that could potentially bias the results (a litter, and an investigator).

Hamsters were infected by 75 metacercariae by gastric intubation (for each out of three liver fluke species) separately at intervals of 3–5 days to avoid cross-bacterial infection. Each time the group of hamsters (24 animals) included animals from several litters. This group had to be divided into two sub-groups (9 animals kept uninfected and 15 animals for the infection). We randomly split up each litter into uninfected and infected sub-groups. This ensured us that a nuisance variable such as a litter could not have been be a source of bias and influenced the outcome. All the procedures were performed aseptically at the SPF facility. The same investigator infected all the animals.

One, two, and three months after the infection, nine uninfected hamsters and five hamsters from each infected groups were euthanized. We performed the sampling of colon fecal and bile from the hepatobiliary system in aseptic conditions after termination of the animals.

All the hamsters were examined daily for signs of illness, injury, or abnormal behavior by SPF trained personnel. Food and water availability and the macroenvironment (temperature, humidity, noise, light intensity, and cleanliness) were also evaluated daily. No unexpected deaths of animals were registered during this study.

### Sample collection and immunohistochemistry

Hamsters were euthanized using carbon dioxide. All the procedures were done aseptically. Bile samples were collected by puncture of the gall bladder. The gastric epithelium was scraped with a scalpel and stored at − 80 °C until use. Colorectal feces were extracted and stored at − 80 °C until use. Adult worms were carefully extracted from bile ducts of a half of the liver. The worms were washed more than 10 times with sterile saline and stored at − 80 °C until use.

For the immunohistochemical analysis, the liver and stomach samples were carefully dissected and placed in 10% buffered formalin (Biovitrum, Russia). After fixation for 3 days, the specimens were dehydrated in a graded series of ethanol solutions and then absolute ethanol, cleared in xylene, and soaked in melted paraffin. After that, the specimens were embedded in paraffin using Microm (Microm, UK). Four-μm-thick slices were prepared by means of a microtome^13,15^. Staining was performed with rabbit antiserum against *H*. *pylori* antibodies (1:100, cat. # ab172611, Abcam, USA), followed by probing with horseradish peroxidase–conjugated secondary antibodies (1:500, cat. # ab205718 Abcam, USA), then staining using the DAB Substrate Kit (Cell Marque, USA). The slices were coverslipped with the VitroGel mounting medium (Biovitrum, Russia) and visualized under an AxioImager A1 microscope (Zeiss) with an AxioCam MRc camera (Zeiss).

### DNA extraction

Feces (150 − 200 mg) and bile (50 − 100 µl) samples were subjected to DNA extraction. The feces were washed with 1 ml of 20% ethanol, homogenized, and then washed twice with 20% ethanol and three times with 1 ml of PBS. The samples were boiled at 100 °C (for 1 min) followed by freeze–thaw cycles using liquid nitrogen and boiling water (4 steps, each for 1 min). The samples were treated with 0.2 mg/ml Proteinase K (Thermo Scientific, USA) for 1 h at 56 °C, and then DNA was extracted by the phenol–chloroform method. Additionally, DNA samples were purified with the DU10 Kit (Biolabmix, Russia).

DNA from the stomach and adult worms was extracted by the standard Proteinase K and phenol–chloroform extraction method. Pieces (15 to 25 mg) of frozen samples of hamster stomachs and individual adult worms were subjected to DNA extraction. DNA concentrations were measured on a Qubit 2.0 spectrophotometer (Invitrogen, USA). The purity of DNA was assessed by measuring absorbance at 260 and 280 nm on a NanoDrop 2000 spectrophotometer (NanoDrop Technologies, Wilmington, DE, USA); ratios of these values ranging from 1.7 to 2.1 were considered acceptable.

### Real-time PCR and quantification of PCR-amplified nucleic acid

Sequences of primers and fluorescently labeled oligonucleotide probes are presented in Supplementary Table S1. *Helicobacter* *pylori* was quantified by quantitative PCR in all DNA samples from four groups of hamsters. The reaction mixture included the EVA Green Master Mix (Synthol Russia). Thermocycling conditions on a CFX96 thermocycler (Bio-Rad, USA) were as follows: 4 min initial denaturation at 95 °C and 40 cycles of 10 s at 95 °C (denaturation), 10 s at 60 °C (annealing), and 10 s at 72 °C (elongation).

Tenfold dilutions of pET plasmid DNA carrying the *H*. *pylori ureA* gene, ranging from 3 × 10^6^ to 3 × 10^2^ copies/PCR, were amplified in triplicate. A calibration curve was built to determine the mean starting amount of DNA in the samples (Supplementary Fig. S1). A range of 3 × 10^6^ to 3 × 10^2^ copies/PCR was found to be optimal because it spanned the exponential portion of the amplification curves, where the amount of an amplified target is directly proportional to the input amount, and gave a linear calibration curve with an R^2^ value of 0.999 (Supplementary Fig. S1).

Gene copy numbers (meaning *H*. *pylori* cell counts) per microgram of DNA were determined by means of the standard curve at a crossing point in a graph of log concentration. The *H*. *pylori* cell concentrations (*ureA* gene amounts) were calculated to ensure the same initial amount of DNA in the samples to be analyzed.

To confirm the presence of bacterial DNA in the DNA samples extracted from adult worms, an RT-PCR assay with primers targeting the V3-V4 region of 16S ribosomal DNA was carried out.

### Statistical analyses

Chi-square (χ^2^) or Fisher’s exact test was performed to compare categorical variables among treatment groups. To compare two groups, nonparametric data were analyzed with the χ^2^ test. The Kruskal–Wallis H test was applied to compare several groups of samples. Data with a *p* value < 0.05 were considered significant.

## Results

### Prevalence of *H*. *pylori* in colorectal feces, stomach, and bile samples of hamsters

Table 1 shows the results of detecting the *H*. *pylori* genes *ureA* and *cagA* in DNA samples extracted from the feces, bile, and the gastric epithelium of hamsters. The *cagA* gene was not detectable in any fecal samples. Therefore, the presence of *H*. *pylori* was determined only by the presence of the *ureA* gene.

**Table 1:** Prevalence of *H*. *pylori* among gastric, stool, and bile samples, as detected by PCR.

	Number of samples	*H. pylori* positive (*ureA*+)	*H. pylori* positive (*ureA*+), %	*cagA*+ positive	Chi-square test	*p* value
**Colorectal feces**						
Uninfected	27	3	11.0	0	ND	ND
*O. felineus*	15	8	53.3	0	8.03	0.004 **
*C. sinensis*	15	11	73.3	0	15.5	0.0001 ***
*O. viverrini*	15	10	66.7	0	12.77	0.0004 ***
**Stomach**						
Uninfected	27	3	11.0	0	ND	ND
*O. felineus*	15	2	13.3	0	0.05	0.83
*C. sinensis*	15	3	20.0	0	0.62	0.43
*O. viverrini*	12	4	33.3	0	2.79	0.09
**Bile**						
Uninfected	5	1	20.0	0	ND	ND
*O. felineus*	6	4	66.7	0	2.4	0.12
*C. sinensis*	6	5	83.3	0	4.41	0.035 *
*O. viverrini*	5	4	80.0	0	3.6	0.057

To compare two groups, nonparametric data were analyzed with the χ2 test. Data with *p* values < 0.05 were considered significant. *A difference between groups (liver fluke–infected vs. uninfected), *p < 0.05; **p < 0.001; ***p < 0.001; ND: not determined.

The prevalence of *H*. *pylori* among fecal samples was significantly higher in the groups of infected animals than in the control group. Fecal *H*. *pylori* was found in 11% of control group animals. The prevalence of fecal *H*. *pylori* was significantly higher in the infected groups than in the control group (χ^2^ = 24.23, df = 3, *p* = 0.000), in particular, this prevalence was 73% among *C*. *sinensis*–infected, 66.7% among *O*. *viverrini*–infected, 53.3% among *O*. *felineus*–infected hamsters (Table 1).

Similar data were obtained from samples of the stomach epithelium and bile of the hamsters (Table 1). In the epithelium of the stomach, *H*. *pylori* DNA was found in three of the 27 uninfected animals (11%). In infected animals, this prevalence was insignificantly higher (χ^2^ = 4.29, df = 3, *p* = 0.23), in particular, it was 20% among *C*. *sinensis*–infected, 33.3% among *O*. *viverrini*–infected, and 13.3% among *O*. *felineus*–infected hamsters (Table 1). In the DNA samples isolated from hamster bile, *H*. *pylori* DNA was detectable in one of 5 samples from the uninfected animals (20%). Among the infected animals, this prevalence was higher (χ^2^ = 6.89, df = 3, *p* = 0.07), in particular, it was 83.3% among *C*. *sinensis*–infected, 80% among *O*. *viverrini*–infected, and 66.7% among *O*. *felineus*–infected hamsters (Table 1).

We did not find any significant differences in the number of *H*. *pylori*–positive samples during the opisthorchiasis infection from 1 to 3 months postinfection. The data are presented in Supplementary Table S2.

The next step was to investigate whether the liver flukes are a reservoir of *H*. *pylori*. We examined 30 DNA samples isolated from 10 worms of each species (*O*. *felineus*, *C*. *sinensis*, and *O*. *viverrini)* of the liver flukes for the presence of the *ureA* and *cagA* genes. We were unable to detect *H*. *pylori* in the trematodes (Table 2). This finding means that none of the three species is a reservoir of *H*. *pylori*. The presence of bacterial DNA in the samples was judged by the presence of PCR results with primers specific to the V3-V4 region of 16S ribosomal DNA. The PCR results indicated that the threshold cycles were 12–14 for *C*. *sinensis*, 14–16 for *O*. *felineus*, and 16–18 for *O*. *viverrini* (Table 2).

**Table 2:** Prevalence of *H*. *pylori* among the adult liver flukes, as detected by PCR after 45 cycles of amplification with primers targeting *H*. *pylori ureA*.

	Number of samples	*H. pylori* positive (*ureA*+)	*H. pylori* positive (*ureA*+), %	*cagA*+	Threshold cycle for 16S rRNA universal primers
*O. felineus*	10	0	0	0	14–16
*C. sinensis*	10	0	0	0	12–14
*O. viverrini*	10	0	0	0	16–18

To compare two groups, nonparametric data were analyzed with the χ2 test. Data with *p* values < 0.05 were considered significant. *A difference between groups (liver fluke–infected vs. uninfected), *p < 0.05; **p < 0.001; ***p < 0.001; ND: not determined.

### Quantification of *H*. *pylori* in colorectal feces, stomach, and bile samples from hamsters by quantitative RT-PCR

Finally, using the efficiency values and calibration curve for the *ureA* gene, the mean amount of input DNA and standard error of the mean were calculated for all cycles during the exponential phase of each amplification curve.

**Figure 1:**
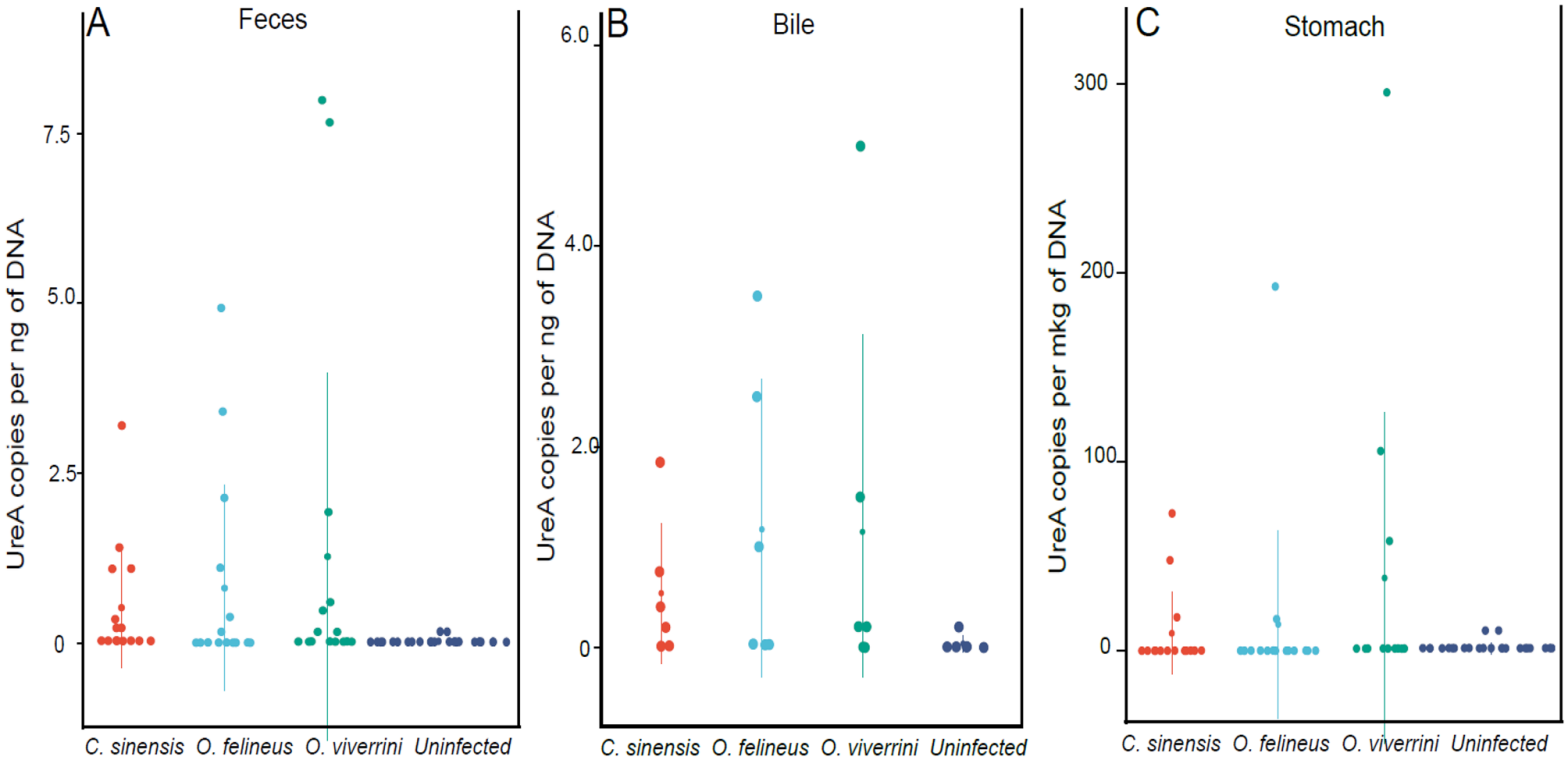
Quantification of *H*. *pylori* by quantitative RT-PCR in colorectal feces, stomach, and bile samples from uninfected hamsters and those infected with one of the liver fluke species. (**A**) Feces samples. (**B**) Bile samples. (**C**) Stomach samples. Tenfold dilutions of the pET plasmid carrying the *H*. *pylori ureA* gene were amplified in triplicate. A linear calibration curve was built to determine the mean starting amount of DNA in each sample and was found to have an R^2^ value of 0.999. Gene copy numbers (meaning *H*. *pylori* cell counts) per microgram of DNA were determined by means of the standard curve at a crossing point in a graph of log concentration. The cell concentration and amounts of DNA were calculated to ensure the same initial DNA amount in each sample to be analyzed.

The mean *ureA* copy number per microgram of DNA was 1601.8 in the feces samples (Fig. 1A) from hamsters infected with *O*. *felineus*, 642.7 in *C*. *sinensis*–infected animals, and 1762.5 in *O*. *viverrini*–infected animals. In contrast to the infected groups, the average number of *ureA* copies per microgram of DNA in the control group was 91.3. The results of the Kruskal–Wallis test [H(3,70) = 21.86, *p* = 0.0001] showed that the number of *ureA* copies in the fecal samples from infected animals significantly differed from that in the uninfected group. We did not find any difference in the prevalence or cell number of *H*. *pylori* among the groups infected with one of the three species of trematodes.

The results on the number of *ureA* gene copies in bile samples were similar (Fig. 1B). The average number of *ureA* gene copies per microgram of DNA was 1762.5 in fecal samples from *O*. *felineus*–infected animals, 645 in the *C*. *sinensis–*infected group, and 1725 in the *O*. *viverrini*–infected group. In contrast to the infected groups, the average number of *ureA* gene copies per microgram of DNA in the control group was 200. The Kruskal–Wallis test [H(3,16) = 5.5, *p* = 0.13] did not reveal statistically significant differences among the groups; apparently, this is due to the small number of *H*. *pylori* positive samples among the samples, in particular in the control group, only in one of 6 samples was the *H*. *pylori* DNA detectable.

The analysis of *ureA* copy numbers per microgram of DNA isolated from hamster stomach samples (Fig. 1C) showed a similar pattern of results as compared to the samples from feces and bile. Namely, in the group of uninfected animals, *H*. *pylori* was found in 2 out of 27 animals, with the number of copies of the *H*. *pylori* gene being ~ 7 per microgram of DNA. In contrast, in the groups of animals infected with *O*. *viverrini*, *C*. *sinensis*, or *O*. *felineus*, the numbers of gene copies were ~ 117, ~ 46, and ~ 106 per microgram of isolated DNA, respectively. It should be noted that the number of *H*. *pylori*–positive stomach samples was not sufficient (2–4 samples) to assess statistical significance of the differences between groups.

### In situ localization of *H*. *pylori*

**Figure 2:**
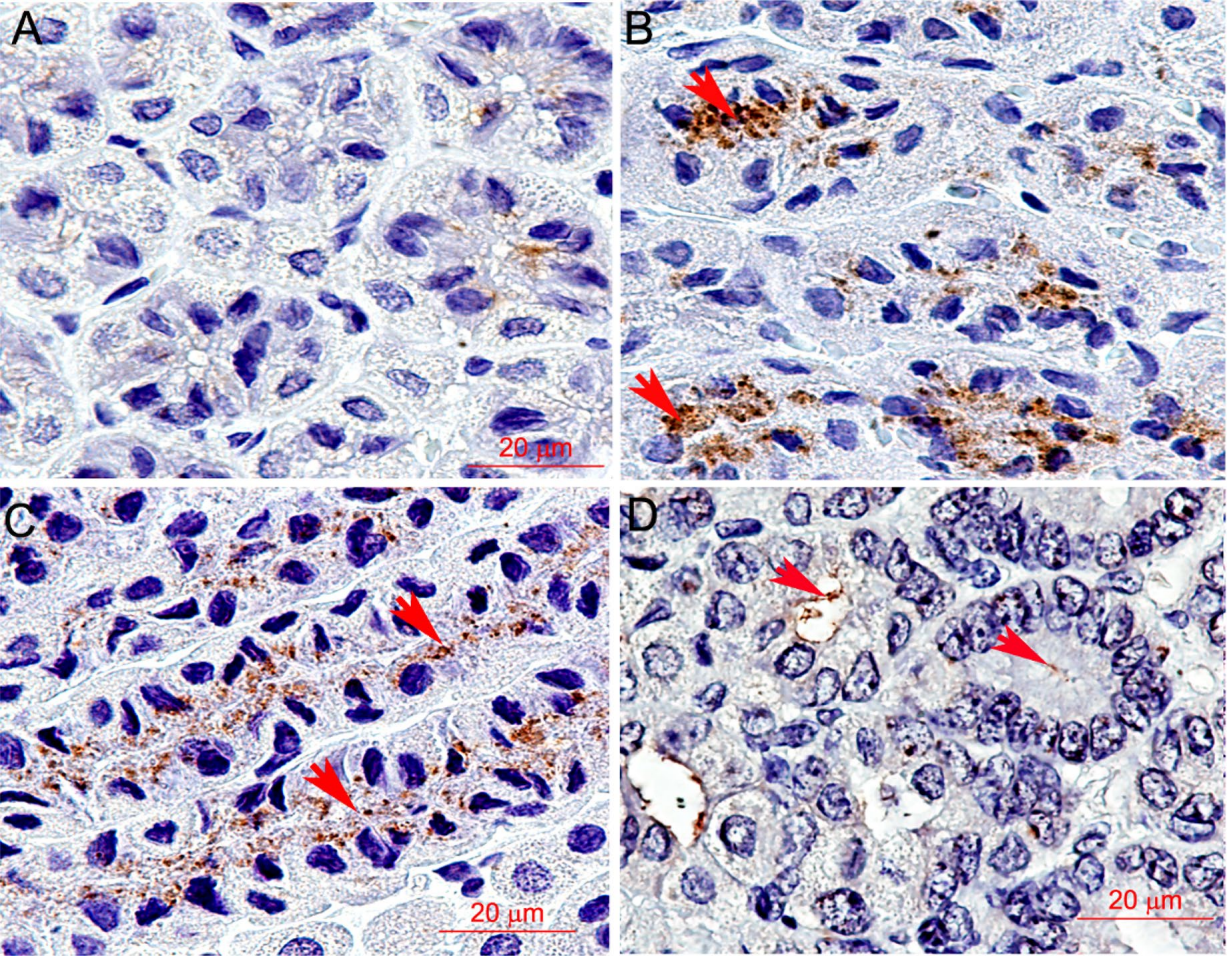
Immunohistochemical *H*. *pylori* signals in the stomach of uninfected hamsters and those infected with one of the liver fluke species. (**A**) An uninfected hamster. (**B**) Infected with *O*. *felineus* liver fluke. (**C**) Infected with *Clonorchis sinensis* liver fluke. (**D**) Infected with *O*. *viverrini* liver fluke. Arrows indicate the staining signal.

To confirm the presence of *H*. *pylori* in the animals, stomachs and livers were examined for *H*. *pylori* content by immunohistochemistry. Representative images of stomach immunohistochemical staining for *H*. *pylori* are presented in Fig. 2. Readers can see that pyloric glands in hamsters from the *O*. *felineus*, *O*. *viverrini*, and *C*. *sinensis* groups stained for this bacterium, whereas in the uninfected group, no staining was observed. Additionally, we examined liver sections for the presence of *H*. *pylori* signals. No *H*. *pylori* signal was detectable in the sections of hamster livers (Supplementary Fig. S2).

## Discussion

Here, a comparative study of *H*. *pylori* DNA content was performed on experimental hamsters infected with one of three trematodes, *O*. *felineus*, *O*. *viverrini*, and *C*. *sinensis*. The main finding of this study is significantly higher prevalence of *H*. *pylori* among colorectal fecal, gastric epithelium, and bile samples of the hamsters infected with any of the three liver flukes. Data on the prevalence of *H*. *pylori* during the infection of mammals with *O*. *felineus* and *C*. *sinensis* were obtained for the first time. The results on the *O*. *viverrini* infection are consistent with the previously obtained data^11,12^. For instance, it was reported that *O*. *viverrini* experimental infection in conventional (non-SPF) status hamsters increases the prevalence of *H*. *pylori* in feces, rectal epithelium, liver tissue, gallbladder tissue, and gastric epithelium samples^11^. Thus, we showed for the first time that any one of the three epidemiologically significant species of liver flukes is capable of increasing the number of *H*. *pylori* cells in the infected host.

The gut microbiota is involved in the homeostasis of the host organism, including the regulation of immune responses and protection against pathogen colonization^16^. The chronic inflammation caused by the colonization of biliary ducts by liver flukes may cause restructuring of the gut microbiota. In particular, infection with the liver fluke *O*. *viverrini* modifies intestinal and biliary microbiomes^17^.

Nonetheless, the influence of helminth infection on microbial diversity of the intestines remains a controversial topic. Studies on experimentally infected individuals indicate an increase in alpha diversity associated with the infection^18^. Moreover, polyparasitism by soil-transmitted nematodes (*Ascaris*, *Trichuris*, and hookworms) increases microbial diversity in the gut; the diversity decreased following deworming^19^. On the contrary, the loss of gut microbial diversity is associated with the development of a number of gastrointestinal disorders^20,21^. It was recently found that an overall reduction in gut microbial alpha diversity, alongside a significant increase in microbial beta diversity, is explained by expanded populations of the phylum Verrucomicrobia and lactobacilli in the gut microbiota of *S*. *mansoni*–infected mice when compared to uninfected mice^22^. A more detailed study revealed that *S*. *mansoni* infection of mice dramatically alters the host intestinal microbiome toward more colitogenic composition^22^. Of note, the colitogenic microbiome is associated with increased abundance levels of genera *Parabacteroides*, *Bacteroides*, and *Helicobacter*^23,24^.

Less is known about how biliary microbiota diversity is related to inflammation in the hepatobiliary system and morbidity. Microbial diversity in the hepatobiliary tract may be lower between an early and advanced stage of primary sclerosing cholangitis, and this diversity is also lower during biliary dysplasia or CCA^9,25^. Moreover, it is known that there is a significant association between CCA and *H*. *pylori* or mixed *H*. *pylori/H*. *bilis* infections in Northeast Thailand^26^. In a hamster model of liver fluke infection–induced biliary disease, there is significantly higher prevalence and severity of coinfection with *H*. *pylori* and *H*. *bilis* (but not *H*. *hepaticus)* in *O*. *viverrini*–infected hamsters compared with uninfected ones, suggesting that the liver fluke may act as a reservoir of *H*. *pylori*^11^.

It is known that some infectious diseases of humans may result from parasite-dwelling bacteria. For example, there is epidemiological evidence of possible interactions between *Salmonella* and *Schistosoma*. *Salmonella* may reside in the tegument and intestinal tracts of all *Schistosoma* species, which are considered multiplication foci for this bacterium^27^. By contrast, our results show that *O*. *felineus*, *O*. *viverrini*, and *C*. *sinensis* are most likely not reservoirs of *H*. *pylori*. In all the fluke samples tested in this work, *H*. *pylori* could not be detected by 45 PCR cycles. This finding can be confirmed by the results indicating the absence of statistically significant differences in the number of *H*. *pylori* gene copies among the groups of animals infected with any one of the three flukes, *O*. *felineus*, *C*. *sinensis*, or *O*. *viverrini*. At the same time, a similar picture was observed when samples of the feces, gastric epithelium, and bile from the infected animals were examined. Thus, the second major result of the study is the absence of interspecies differences in the effect of a liver fluke infection on the prevalence of *H*. *pylori* and on the number of cells of this pathogen in the host.

All the hamsters used in the study had SPF status and were kept during the experiments at the SPF animal facility, suggesting that the animals did not have major known infections, including *H*. *pylori*. Indeed, by a standard PCR assay that includes 35 cycles of amplification, it was not possible to detect *H*. *pylori* in the uninfected animals here. On the other hand, with an increase in the number of PCR cycles to 40, *H*. *pylori* was detected in samples of feces, bile, and gastric epithelium in 3 of the 27 animals of the uninfected group. We assume that a minor reservoir of *H*. *pylori* infection was in the microbiome of uninfected animals. After the liver fluke infection, this reservoir might have been a source of bacteria expanding and spreading throughout the body. After quantitative analysis, it turned out that *H*. *pylori* gene copy numbers in the uninfected animals were extremely low: ~ 90 copies per microgram of isolated DNA, whereas in the infected samples, this figure reached 5000–7000 gene copies per microgram of DNA. These results mean that the liver fluke infections can cause changes in a host’s microbial populations, in particular a significant increase in *H*. *pylori* abundance.

Some *H*. *pylori* strains have the *cagA* gene, which encodes one of the most important virulence factors^28,29^. In our study, we did not find this gene in any of the samples from either the control or infected groups. This indicates that *cagA*-positive *H*. *pylori* was not present in the experimental animals, not even a single cell.

It has previously been demonstrated that there is higher prevalence of *H*. *pylori* among people with opisthorchiasis than among people without opisthorchiasis. Data on frequent coinfection with *H*. *pylori* and *O*. *viverrini* are convincing^30^. In the geographic areas of opisthorchiasis felinea, such studies have not been carried out, but there is evidence that the clinical course of peptic ulcer disease (possibly associated with *H*. *pylori)*, when comorbid with chronic opisthorchiasis, differs from the usual one. It was found that when concurrent with chronic opisthorchiasis, persistent functional disorders of the gastroduodenal system are characterized by long duration of ulcer healing with prolonged exacerbations. The average duration and scarring of the ulcer are significantly lengthened in comorbidity cases, and complete clinical and endoscopic remission of peptic ulcer disease does not occur during chronic opisthorchiasis^31,32^. Most authors have noted that a necessary condition for successful treatment of mixed disorders of the stomach and duodenum is anthelmintic therapy, which helps to prevent inflammatory changes in the gastroduodenal mucosa^32–36^. These data also indicate a tight connection between *H*. *pylori* and *O*. *felineus* infections.

How can the liver fluke infections affect the mammalian microbiota and increase the prevalence of *H*. *pylori*? Liver flukes excrete and/or secrete mediators^37^ and bacteria from their gut, thereby altering host’s liver functions that may modify the biliary environment and which in turn may alter the microbiota. It is also likely that chronic inflammation during infection^13,15^ or complex dysfunctions of metabolic and immune systems of the host^16,38^ can contribute to significant changes in a local and possibly total microbiome.

One of the causes of high frequency of *Helicobacter* infection among opisthorchiasis-infected individuals may be cholestasis, which ultimately leads to higher risk of fecal and biliary infection. *Helicobacter* spp. have been detected previously in the stomach, liver, gallbladder, and colon mucosa^39^. The findings presented here revealed the infection of the liver and gallbladder with *H*. *pylori* and indicate that the *H*. *pylori* in these tissues originates from the microbiota of the hamster stomach or colon rather than from the liver fluke.

How can the microbiota contribute to liver pathology? It is known that *Helicobacter* in naturally infected hamsters^26,39,40^ is associated with hepatobiliary disorders, like chronic hepatitis, hepatic dysplasia, fibrosis, and biliary hyperplasia by 2 years of age. There is a hypothesis explaining how dysfunctions of the gut bacterial microbiota affect liver diseases. Nonetheless, it has long been known that cholelithiasis, cholecystitis, and cholangitis in humans are associated with bacterial colonization of bile^41,42^. Quantitative and qualitative disturbances of the microbiome are an increasingly recognized contributing factor of chronic liver diseases^43^. In healthy individuals, strict separation of microbial cells from a host compartment forms the basis of a symbiotic relationship between the host and microbiota^7^. An abnormal bacterial gut microbiota can promote disorders not only via local effects but also at distant sites such as the liver, heart, brain, and the hematopoietic system^7^.

The involvement of microbiota alternations, in particular *H*. *pylori* abundance changes, in the development of severe complications of opisthorchiasis or clonorchiasis in humans cannot be ruled out. *Helicobacter* *pylori* infection was the first bacterial infection implicated in gastrointestinal diseases including gastric adenocarcinoma^44^. The strong association between liver fluke and *H*. *pylori* infections reported in previous studies is consistent with a role of this *Helicobacter* species in the hepatobiliary disorders characteristic of opisthorchiasis^26, 40^. Moreover, serological assays point to active infection with *H*. *pylori* and *H*. *bilis* in Thais at high risk of CCA^26^. There is no doubt that the presence of opisthorchiasis indicates an increase in the prevalence of *H*. *pylori* infection. The results of our study, namely a relation between liver fluke infection (any of the three species) and the expansion of *H*. *pylori* in the gastrointestinal tract, can help to improve the methods of diagnosis and treatment of the respective human illnesses.

In particular, an increase of the *H*. *pylori* prevalence can be utilized in the diagnosis of opisthorchiasis, clonorchiasis, or *H*. *pylori*–associated gastritis, gastric ulcer, and duodenal ulcer. For example, a sharp increase in a patient’s markers of *H*. *pylori* infection and presentation of symptoms indicating *H*. *pylori*–associated gastrointestinal diseases suggest that the patient may have opisthorchiasis or clonorchiasis. Furthermore, if a patient with opisthorchiasis or clonorchiasis begins to develop symptoms or signs of gastritis, gastric ulcer, or duodenal ulcer, it can be assumed that there is an increase in the prevalence of *H*. *pylori* in the patient’s body. It is important to note that when a coinfection by a fluke and *H*. *pylori* is found and one of them was detected earlier than the other, it is necessary to adjust the treatment with anthelmintic and anti-*Helicobacter* drugs and to monitor the two diseases simultaneously. Moreover, decreased signs of the disease caused by *H*. *pylori* may indicate successful elimination of trematodes.

Our findings appear to be novel in terms of comparative assessment of the host microbiome and *Helicobacter* abundance during infections with three epidemiologically important liver fluke species. Overall, infection with *O*. *felineus*, *C*. *sinensis*, or *O*. *viverrini* liver flukes significantly modified biliary and colon microbiomes in this study, by increasing *H*. *pylori* abundance. It will be worthwhile to investigate this phenomenon further, including in residents of the regions where liver flukes are endemic and where such infections increase the risk of bile duct cancer. Further research will enable a deeper look at the mechanisms behind the connection between trematodes and *H*. *pylori*, thus allowing to develop preventive measures against the coinfection.

### Ethics approval

The experiments were performed in compliance with the EU Directive 2010/63/EU for animal experiments and with the ARRIVE guidelines. Study design protocols and standard operating procedures (concerning the hamsters and the fish) were approved by the Committee on the Ethics of Animal Experiments at the ICG SB RAS (permit number 42 of 25 May 2018).

## Supplementary Information

Supplementary Information.

## Data Availability

All data generated or analyzed during this study are included in this published article.

## Abbreviations

*O*. *felineus*: *Opisthorchis felineus*
*C*. *sinensis*: *Clonorchis sinensis*
*O*. *viverrini*: *Opisthorchis viverrini*
*H*. *pylori*: *Helicobacter pylori*
CCA: Cholangiocarcinoma

## References

[1] Petney TN, Andrews RH, Saijuntha W, Wenz-Mücke A, Sithithaworn P (2013). The zoonotic, fish-borne liver flukes *Clonorchis sinensis, Opisthorchis felineus* and *Opisthorchis viverrini*. Int. J. Parasitol..

[2] Pakharukova MY, Correia da Costa JM, Mordvinov VA (2019). The liver fluke *Opisthorchis felineus* as a group III or group I Carcinogen. 4open.

[3] Lun ZR, Gasser RB, Lai DH, Li AX, Zhu XQ, Yu XB (2005). Clonorchiasis: a key foodborne zoonosis in China. Lancet Infect. Dis..

[4] IARC Working Group on the Evaluation of Carcinogenic Risks to Humans, Biological agents. Volume 100 B. A review of human carcinogens. *IARC Monogr. Eval. Carcinog. Risks Hum.* **100** Pt B, 1–441 (2012). PMC4781184 23189750

[5] Fedorova OS, Fedotova MM, Zvonareva OI, Mazeina SV, Kovshirina YV, Sokolova TS, Golovach EA, Kovshirina AE, Konovalova UV, Kolomeets IL, Gutor SS, Petrov VA, Hattendorf J, Ogorodova LM, Odermatt P (2020). *Opisthorchis felineus* infection, risks, and morbidity in rural Western Siberia, Russian Federation. PLoS Negl. Trop Dis..

[6] Saltykova IV, Petrov VA, Brindley PJ (2018). Opisthorchiasis and the Microbiome. Adv. Parasitol..

[7] Yu LX, Schwabe RF (2017). The gut microbiome and liver cancer: mechanisms and clinical translation. Nat. Rev. Gastroenterol. Hepatol..

[8] Zhou D, Zhang Y, Gong W, Mohamed SO, Ogbomo H, Wang X, Liu Y, Quan Z (2011). Are *Helicobacter pylori* and other Helicobacter species infection associated with human biliary lithiasis? A meta-analysis. PLoS ONE.

[9] Pereira P, Aho V, Arola J, Boyd S, Jokelainen K, Paulin L, Auvinen P, Farkkila M (2017). Bile microbiota in primary sclerosing cholangitis: impact on disease progression and development of biliary dysplasia. PLoS ONE.

[10] Murphy G, Michel A, Taylor PR, Albanes D, Weinstein SJ, Virtamo J, Parisi D, Snyder K, Butt K, McGlynn KA, Koshiol J, Pawlita M, Lai GY, Abnet CC, Dawsey SD, Freedman ND (2014). Association of seropositivity to Helicobacter species and biliary tract cancer in the ATBC study. Hepatology.

[11] Deenonpoe R, Chomvarin C, Pairojkul C, Chamgramol Y, Loukas A, Brindley PJ, Sripa B (2015). The carcinogenic liver fluke *Opisthorchis viverrini* is a reservoir for species of Helicobacter. Asian Pac. J. Cancer Prev..

[12] Deenonpoe R, Mairiang E, Mairiang P, Pairojkul C, Chamgramol Y, Rinaldi G, Loukas A, Brindley PJ, Sripa B (2017). Elevated prevalence of Helicobacter species and virulence factors in opisthorchiasis and associated hepatobiliary disease. Sci. Rep..

[13] Pakharukova MY, Zaparina OG, Kapushchak YK, Baginskaya NV, Mordvinov VA (2019). *Opisthorchis felineus* infection provokes time-dependent accumulation of oxidative hepatobiliary lesions in the injured hamster liver. PLoS ONE.

[14] Pakharukova MY, Samsonov VA, Serbina EA, Mordvinov VA (2019). A study of tribendimidine effects *in vitro* and *in vivo* on the liver fluke *Opisthorchis felineus*. Parasit Vectors.

[15] Pakharukova MY, Zaparina OG, Kovner AV, Mordvinov VA (2019). Inhibition of *Opisthorchis felineus* glutathione-dependent prostaglandin synthase by resveratrol correlates with attenuation of cholangiocyte neoplasia in a hamster model of opisthorchiasis. Int. J. Parasitol..

[16] Pickard JM, Zeng MY, Caruso R, Núñez G (2017). Gut microbiota: Role in pathogen colonization, immune responses, and inflammatory disease. Immunol. Rev..

[17] Plieskatt JL, Deenonpoe R, Mulvenna JP, Krause L, Sripa B, Bethony JM, Brindley PJ (2013). Infection with the carcinogenic liver fluke *Opisthorchis viverrini* modifies intestinal and biliary microbiome. FASEB J..

[18] Giacomin P, Zakrzewski M, Jenkins TP, Su X, Al-Hallaf R, Croese J, de Vries S, Grant A, Mitreva M, Loukas A (2016). Changes in duodenal tissue-associated microbiota following hookworm infection and consecutive gluten challenges in humans with coeliac disease. Sci. Rep..

[19] Lee SC, Ngui R, Tan TK, Muhammad Aidil R, Lim YA (2014). Neglected tropical diseases among two indigenous subtribes in peninsular Malaysia: highlighting differences and co-infection of helminthiasis and sarcocystosis. PLoS ONE.

[20] Imhann F, Vich Vila A, Bonder MJ, Fu J, Gevers D, Visschedijk MC, Spekhorst LM, Alberts R, Franke L, van Dullemen HM (2018). Interplay of host genetics and gut microbiota underlying the onset and clinical presentation of inflammatory bowel disease. Gut.

[21] Lapthorne S, Pereira-Fantini PM, Fouhy F, Wilson G, Thomas SL, Dellios NL, Scurr M, O’Sullivan O, Ross RP, Stanton C (2013). Gut microbial diversity is reduced and is associated with colonic inflammation in a piglet model of short bowel syndrome. Gut Microb..

[22] Floudas A, Aviello G, Schwartz C, Jeffery IB, O'Toole PW, Fallon PG (2019). *Schistosoma mansoni* Worm infection regulates the intestinal microbiota and susceptibility to colitis. Infect Immun..

[23] Dziarski R, Park SY, Kashyap DR, Dowd SE, Gupta D (2016). Pglyrp-regulated gut microflora *Prevotella falsenii, Parabacteroides distasonis* and *Bacteroides eggerthii* enhance and *Alistipes finegoldii* attenuates colitis in mice. PLoS ONE.

[24] Coccia M, Harrison OJ, Schiering C, Asquith MJ, Becher B, Powrie F, Maloy KJ (2012). IL-1beta mediates chronic intestinal inflammation by promoting the accumulation of IL-17A secreting innate lymphoid cells and CD4 Th17 cells. J. Exp. Med..

[25] Pereira SG, Moura J, Carvalho E, Empadinhas N (2017). Microbiota of chronic diabetic wounds: ecology, impact, and potential for innovative treatment strategies. Front. Microbiol..

[26] Boonyanugomol W, Chomvarin C, Sripa B, Bhudhisawasdi V, Khuntikeo N, Hahnvajanawong C, Chamsuwan A (2012). *Helicobacter pylori* in Thai patients with cholangiocarcinoma and its association with biliary inflammation and proliferation. HPB (Oxford).

[27] Ashour DS, Othman AA (2020). Parasite-bacteria interrelationship. Parasitol. Res..

[28] Cid TP, Fernandez MC, Benito Martinez S, Jones NL (2013). Pathogenesis of *Helicobacter pylori* infection. Helicobacter.

[29] Hatakeyama M (2014). *Helicobacter pylori* CagA and gastric cancer: a paradigm for hit-and-run carcinogenesis. Cell Host Microbe.

[30] Dangtakot R, Pinlaor S, Itthitaetrakool U, Chaidee A, Chomvarin C, Sangka A, Wilailuckana C, Pinlaor P (2017). Coinfection with *Helicobacter pylori* and *Opisthorchis viverrini* enhances the severity of hepatobiliary abnormalities in hamsters. Infect. Immun..

[31] Beloborodova E.I., Pavlenko, O.A., Tsygolnik, M.D. *Peptic ulcer on the background of chronic opisthorchiasis*. (Tomsk State University of Control Systems and Radioelectronics, 1997). (Russian)

[32] Pavlenko OA (1988). Diagnostics of duodenogastric reflux in patients with peptic ulcer disease in combination with chronic opisthorchiasis. Med. Parasitol. Parasitic Dis..

[33] Beloborodova, E.I., Zadorozhnaya, N.A., Tsygolnik, M.D. *Diagnostics and treatment of duodenitis against the background of chronic opisthorchiasis*. (Tomsk, 1998). (Russian)

[34] Sadkova TN, Deryabina MS, Komarova NM (1973). Damage to the gastroduodenal system in opisthorchiasis. Med. Bus..

[35] Yablokov, D.D. *Human opisthorchiasis*. (Tomsk State University, 1979). (Russian)

[36] Kolosovskaya TA, Beloborodova EI, Kalyuzhina MI, Tilichenko YA, Kalyuzhin VV, Pavlenko OA (1997). Clinical and functional state of the stomach in patients in the residual phase of chronic opisthorchiasis. Sib. J. Gastroenterol. Hepatol..

[37] Gouveia MJ, Pakharukova MY, Laha T, Sripa B, Maksimova GA, Rinaldi G, Brindley PJ, Mordvinov VA, Amaro T, Santos LL, Costa JMCD, Vale N (2017). Infection with *Opisthorchis felineus* induces intraepithelial neoplasia of the biliary tract in a rodent model. Carcinogenesis.

[38] Cortés A, Peachey L, Scotti R, Jenkins TP, Cantacessi C (2019). Helminth-microbiota cross-talk—a journey through the vertebrate digestive system. Mol. Biochem. Parasitol..

[39] Fox JG, Shen Z, Muthupalani S, Rogers AR, Kirchain SM, Dewhirst FE (2009). Chronic hepatitis, hepatic dysplasia, fibrosis, and biliary hyperplasia in hamsters naturally infected with a novel Helicobacter classified in the *H. bilis* cluster. J. Clin. Microbiol..

[40] Boonyanugomol W, Chomvarin C, Sripa B, Chau-In S, Pugkhem A, Namwat W, Wongboot W, Khampoosa B (2012). Molecular analysis of *Helicobacter pylori* virulent associated genes in hepatobiliary patients. HPB (Oxford).

[41] Carpenter HA (1998). Bacterial and parasitic cholangitis. Mayo Clin. Proc..

[42] Maluenda F, Csendes A, Burdiles P, Diaz J (1989). Bacteriological study of choledochal bile in patients with common bile duct stones, with or without acute suppurative cholangitis. Hepatogastroenterology..

[43] Ponziani FR, Bhoori S, Castelli C, Putignani L, Rivoltini L, Del Chierico F, Sanguinetti M, Morelli D, Paroni Sterbini F, Petito V, Reddel S, Calvani R, Camisaschi C, Picca A, Tuccitto A, Gasbarrini A, Pompili M, Mazzaferro V (2019). Hepatocellular carcinoma is associated with gut microbiota profile and inflammation in nonalcoholic fatty liver disease. Hepatology.

[44] Bouvard V, Baan R, Straif K, Grosse Y, Secretan B, El Ghissassi F, Benbrahim-Tallaa L, Guha N, Freeman C, Galichet L, Cogliano V (2009). A review of human carcinogens—part B: biological agents. Lancet Oncol..

