# Supplementary material for: A comparative study of Helicobacter pylori infection in hamsters experimentally infected with liver flukes Opisthorchis felineus, Opisthorchis viverrini, or Clonorchis sinensis
Source: Sci Rep. 2021 Apr 8;11:7789. doi: 10.1038/s41598-021-87446-x (PMC8032737; doi:10.1038/s41598-021-87446-x)
Supplement: Supplementary file 1 — Supplementary Information. [file 41598_2021_87446_MOESM1_ESM.pdf]

A comparative study of *Helicobacter pylori* infection in hamsters experimentally infected with liver flukes *Opisthorchis felineus*, *Opisthorchis viverrini*, or *Clonorchis sinensis*

Maria Y. Pakharukova, Oxana Zaparina, Sung-Jong Hong, Banchob Sripa, Viatcheslav A. Mordvinov

Supplementary Table S1. Primer sequences for the detection of species of Helicobacter

Primer	Sequences
UreA_F	GCGCA CTCTT TTAAA ACCGG
UreA_R	ACGAG TTTTG TCCCA TCAGG
UreA_probe	VIC-TCATG CTTGC CACGC CATCC-BHQ2
CagA_F	CCCCA TGTCC AACCA GATAT AG
CagA_R	GCGAC TCCCT CAACA TCTAA C
CagA_probe	VIC-TCAAG CAAAT CCCTA GATTC AGGG G-BHQ2
16S_0341_F	CCTAC GGGNG GCWGC AG
16S_0785_R	GACTA CHVGG GTATC TAATC C

Supplementary Table S2. Prevalence of *H. pylori* in gastric, stool, and bile samples does not depend on duration of the liver fluke infection.

**Stool samples**								
	Uninfected		*O. felineus*		*C. sinensis*		*O. viverrini*	
Duration of infection	Number of samples	*H. pylori* +	Number of samples	*H. pylori* +	Number of samples	*H. pylori* +	Number of samples	*H. pylori* positive
1 month	9	0	5	4	5	2	5	5
2 months	9	3	5	2	5	5	5	3
3 months	9	0	5	2	5	3	5	3
**Stomach samples**								
	Uninfected		*O. felineus*		*C. sinensis*		*O. viverrini*	
Duration of infection	Number of samples	*H. pylori* positive	Number of samples	*H. pylori* positive	Number of samples	*H. pylori* positive	Number of samples	*H. pylori* positive
1 month	9	0	5	0	5	1	5	1
2 months	9	3	5	0	5	0	5	2
3 months	9	0	5	2	5	2	5	1
**Bile samples**								
	Uninfected		*O. felineus*		*C. sinensis*		*O. viverrini*	
Duration of infection	Number of samples	*H. pylori* positive	Number of samples	*H. pylori* positive	Number of samples	*H. pylori* positive	Number of samples	*H. pylori* positive
1 month	0	0	2	1	2	2	0	0
2 months	4	1	1	1	1	1	3	2
3 months	1	0	3	2	3	2	2	2

Prevalence of *H. pylori* in gastric, stool, and bile samples was detected by quantitative PCR.

Supplementary Figure S1.

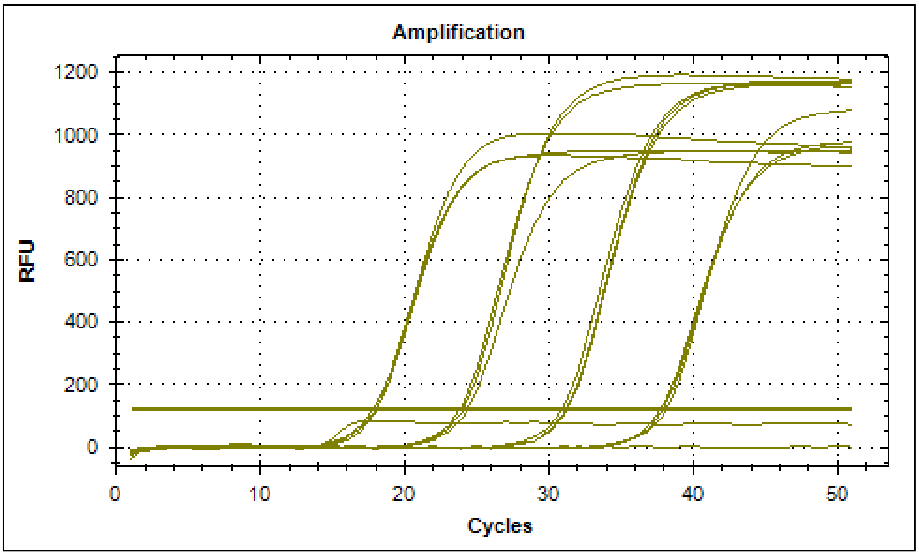

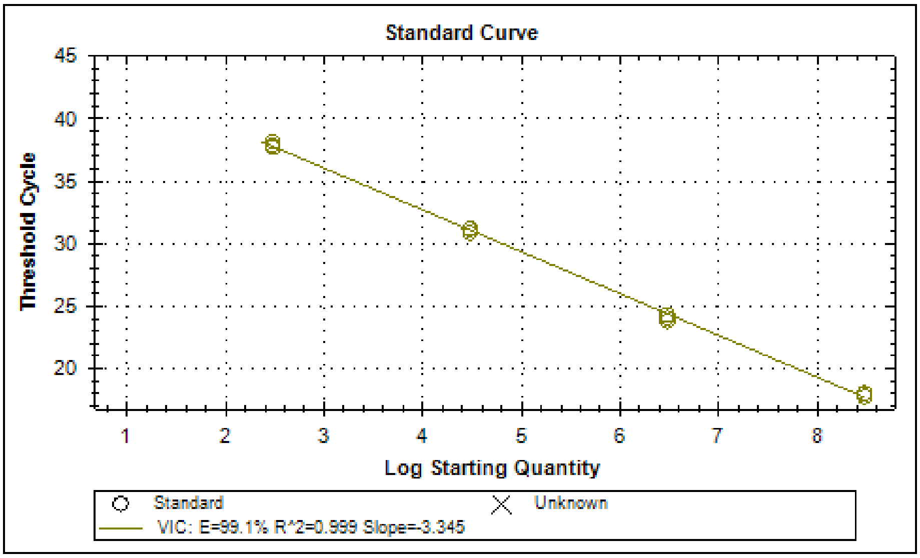

Quantification of PCR and standard curve construction

Ten-fold dilutions of plasmid DNA with target *ureA* gene, ranging from $3 \times 10^6 - 3 \times 10^2$ copies/PCR, were amplified in triplicate. The calibration curve was built using CFX Manager 2.1 (Bio-Rad).

A range of $3 \times 10^6 - 3 \times 10^2$ copies/PCR, was optimal, as this spanned the exponential portion of the amplification curves, where the amount of amplified target is directly proportional to the input amount, and gave a linear calibration curve with R^2 value of 0.999.

Supplementary Figure S2.

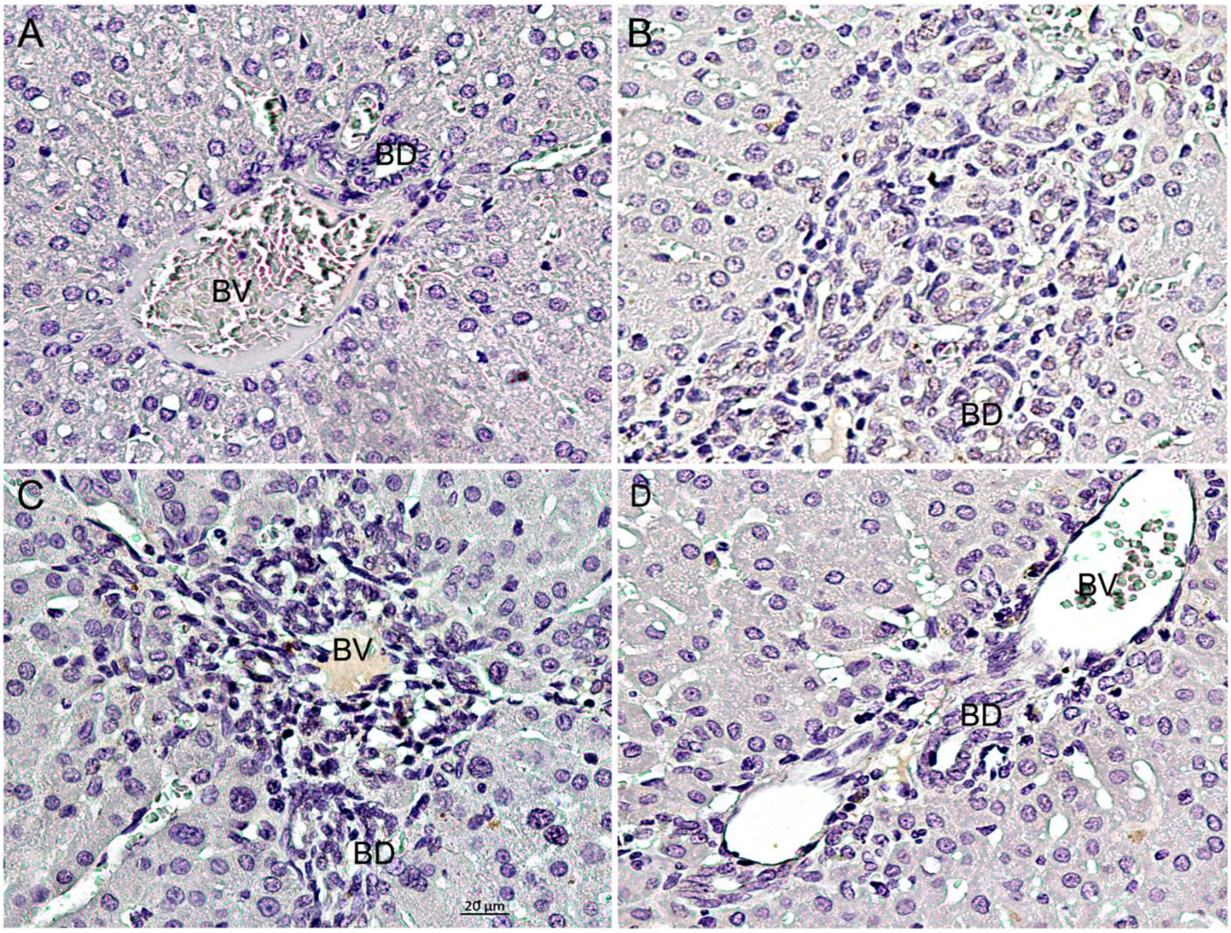

The absence of *H. pylori* specific signal in the liver of uninfected hamsters and infected with one of the liver fluke species. A. Uninfected. **B.** Infected with *O. felineus* liver fluke. **C**. Infected with *O. viverrini* liver fluke. **D.** Infected with *Clonorchis sinensis* liver fluke. BV: blood vessel; BD: bile ducts
